# Synergistic effects of high dietary calcium and exogenous parathyroid hormone in promoting osteoblastic bone formation in mice

**DOI:** 10.1017/S0007114514004309

**Published:** 2015-03-06

**Authors:** Yuxu Feng, Min Zhou, Qunhu Zhang, Huan Liu, Yong Xu, Lei Shu, Jue Zhang, Dengshun Miao, Yongxin Ren

**Affiliations:** 1 Department of Orthopaedics, The First Affiliated Hospital, Nanjing Medical University, Nanjing, Jiangsu, People's Republic of China; 2 Department of Geriatric Endocrinology, Jinling Hospital, Nanjing University School of Medicine, Nanjing, People's Republic of China; 3 Department of Emergency, The Affiliated Jiangning Hospital of Nanjing Medical University, Nanjing, Jiangsu, People's Republic of China; 4 Center for Bone and Stem Cell Research, Nanjing Medical University, Nanjing, Jiangsu, People's Republic of China

**Keywords:** Parathyroid hormone, High-calcium diet, Osteoblasts, Osteoclasts, Synergistic effects, Bone formation

## Abstract

In the present study, we investigated whether high dietary Ca and exogenous parathyroid hormone 1–34 fragments (PTH 1–34) have synergistic effects on bone formation in adult mice, and explored the related mechanisms. Adult male mice were fed a normal diet, a high-Ca diet, a PTH-treated diet, or a high-Ca diet combined with subcutaneously injected PTH 1–34 (80 μg/kg per d) for 4 weeks. Bone mineral density, trabecular bone volume, osteoblast number, alkaline phosphatase (ALP)- and type I collagen-positive areas, and the expression levels of osteoblastic bone formation-related genes and proteins were increased significantly in mice fed the high-Ca diet, the PTH-treated diet, and, even more dramatically, the high-Ca diet combined with PTH. Osteoclast number and surface and the ratio of receptor activator for nuclear factor-κB ligand (RANKL):osteoprotegerin (OPG) were decreased in the high-Ca diet treatment group, increased in the PTH treatment group, but not in the combined treatment group. Furthermore, third-passage osteoblasts were treated with high Ca (5 mm), PTH 1–34 (10^− 8^ m) or high Ca combined with PTH 1–34. Osteoblast viability and ALP activity were increased in either the high Ca-treated or PTH-treated cultures and, even more dramatically, in the cultures treated with high Ca plus PTH, with consistent up-regulation of the expression levels of osteoblast proliferation and differentiation-related genes and proteins. These results indicate that dietary Ca and PTH play synergistic roles in promoting osteoblastic bone formation by stimulating osteoblast proliferation and differentiation.

Parathyroid hormone (PTH) is secreted by parathyroid chief cells, which plays a key role in bone and kidney metabolism. Mature PTH consists of eighty-four amino acid residues, and its biological activity is predominantly determined by the N-terminal amino acid sequence from 1 to 34^(^ ^1^ ^)^. Its 1–34 fragment, namely PTH 1–34, is currently in clinical use. The effects of PTH 1–34 on bone depend on the dose and mode of administration. Numerous animal experiments and human studies have shown that intermittent injection of PTH 1–34 exerts anabolic effects by increasing bone mass (BM), whereas continuous PTH 1–34 treatment decreases the BM^(^ ^2^ ^)^. Studies^(^ ^3^ ^)^ have shown that intermittent PTH can promote bone formation by a variety of mechanisms including: (1) promoting pre-osteoblast differentiation in osteoblasts; (2) suppressing adipocyte formation; (3) changing bone lining cells into active osteoblasts; (4) inhibiting osteoblast apoptosis; (5) playing an indirect role through autocrine/paracrine molecules, including insulin-like growth factor 1 (IGF-1), Wnt and fibroblast growth factors. However, the specific molecular mechanism remains unclear and requires further in-depth study.

Human recombinant PTH 1–34 (teriparatide) is the only currently recommended agent that promotes bone formation, and was approved by the US Food and Drug Administration in 2000 for the treatment of postmenopausal women with osteoporosis who are at high risk for fracture^(^ ^4^ ^)^. However, its curative effect is not ideal when used alone, because PTH can promote bone formation as well as stimulate bone absorption. In early 1993, Shen *et al.* ^(^ ^5^ ^)^ found that PTH combined with oestrogen can effectively increase trabecular BM and connections. In a clinical study, Cosman *et al.* ^(^ ^6^ ^)^ also found that the PTH/oestrogen combination can significantly improve BM and bone mineral density (BMD) in patients, simultaneously reducing the risk of vertebral fracture. Thus, using PTH in combination with other drugs to promote bone formation is another target for research.

Studies have found that high concentrations of Ca ions can cause osteoclast apoptosis^(^ ^7^ ^)^ as well as function as a chemical chemokine to promote osteoblast precursors to move to bone absorption sites, and stimulate osteoblast proliferation and differentiation to produce new bone^(^ ^8^ ^,^ ^9^ ^)^. These findings suggest that Ca may be an important coupling factor between bone resorption and bone formation by inhibiting bone resorption and stimulating bone formation. Previous studies have suggested that high concentrations of Ca ions inhibit bone resorption, by inhibiting the generation of endogenous PTH and 1,25-dihydroxyvitamin D_3_^(^ ^10^ ^)^. A recent *in vitro* study has found that high concentrations of Ca could activate the mitogen-activated protein kinase (MAPK) pathway mediated by Ca-sensing receptor (CaSR) to stimulate osteoblast proliferation and differentiation^(^ ^8^ ^)^. Our previous studies^(^ ^11^ ^–^ ^14^ ^)^ have indicated that the effect of endogenous PTH on bone formation or absorption might depend on the concentration of extracellular Ca ions. Exogenous PTH affects endochondral bone formation, at least partially, by increasing the extracellular Ca concentration to stimulate indirectly chondrocyte and osteoblast proliferation and differentiation. Recent research has also shown that PTH and Ca can each exert cooperative effects on osteoblastic bone formation in the neonate^(^ ^15^ ^)^.

Based on the aforementioned data, high Ca and exogenous PTH play a synergistic role in promoting the bone formation process, focusing mainly on osteoblast proliferation and differentiation. However, it is still unclear whether the combination of high Ca and PTH may play a synergistic role during bone formation. To answer this question and identify related mechanisms, we fed adult mice a high-Ca diet and treated them with exogenous PTH 1–34 *in vivo*. The alterations of bone formation and resorption parameters were examined using radiological, histological, and cellular and molecular biological methods. Furthermore, we cultured osteoblasts in the presence or absence of a high concentration of Ca and PTH 1–34 *in vitro*, and examined their effects on the proliferation and differentiation of osteoblasts.

## Materials and methods

### Animals

Male C57BL/6J mice (4 weeks of age) were purchased from the Experimental Animal Center of Nanjing Medical University. Animals were housed five per cage in a micro-isolator room on a 12 h light–12 h dark cycle in the University Animal Center. Beginning at 6 weeks of age, mice were fed purified rodent diets from Harlan Teklad that were identical in content except that they contained either a normal Ca (1 % Ca^2+^) or a high Ca (2 % Ca^2+^) level. Simultaneously, mice received daily injections of vehicle or PTH 1–34 (80 μg/kg per d). After 4 weeks of treatment, mice were used for experiments as described. The Institutional Animal Care and Use Committee of Nanjing Medical University approved all experiments (approval ID 2008-00 518).

### Radiography and bone mineral density measurement

For radiography, the left tibias from 10-week-old mice were removed and dissected free of soft tissue, and fixed overnight in 70 % ethanol. X-ray images were obtained using a Faxitron machine (Model 805; Faxitron X-ray Corporation) under constant conditions (22 kV, 4 min exposure), and using a Kodak X-Omat TL film (Eastman Kodak Company). For the measurement of tibial BMD, a PIXImus densitometer (Lunar PIXImus Corporation) was used (5 min image acquisition with a precision of 1 % CV for skeletal BMD). The PIXImus software automatically calculated the BMD and recorded data in Microsoft Excel files (Microsoft Corporation).

### Micro-computed tomography

After radiography and BMD measurement, the left tibias were analysed sequentially, as described previously, using micro-computed tomography with a Skyscan 1072 scanner and associated analysis software (Skyscan)^(^ ^16^ ^)^. Briefly, image acquisition was performed at 100 kV and 98 mA with a 0·9° rotation between frames. During scanning, samples were enclosed in tightly fitting plastic wrap to prevent movement and dehydration. Thresholding was applied to the images to segment bone from background. Two-dimensional images were used to generate three-dimensional renderings using the 3D Creator software supplied with the instrument (Sky Scan). The resolution of the micro-computed tomography images is 18·2 mm.

### Histology

The right tibias were removed from mice, fixed overnight at 4°C in PLP fixative (2 % paraformaldehyde containing 0·075 m-lysine and 0·01 m-sodium periodate), and histologically processed as described previously^(^ ^17^ ^)^. The tibias were decalcified in EDTA and glycerol solution for 5–7 d at 4°C. The decalcified tibias were dehydrated and embedded in paraffin, after which 5 mm sections were cut on a rotary microtome. The sections were stained with haematoxylin and eosin, or histochemically for total collagen and alkaline phosphatase (ALP) activity and tartrate-resistant acid phosphatase (TRAP), or for immunohistochemical staining as described below.

### Collagen, alkaline phosphatase and tartrate-resistant acid phosphatase histochemical staining

Total collagen was detected in paraffin-embedded sections using a modified version of Lopez-De Leon & Rojkind's^(^ ^18^ ^)^ method as described previously. Dewaxed sections were exposed to 1 % Sirius red in saturated picric acid for 1 h. After washing with distilled water, sections were counterstained with haematoxylin and mounted with Biomount medium.

Enzyme histochemical analysis was performed for the determination of ALP activity, as described previously^(^ ^19^ ^)^. Briefly, following pre-incubation overnight with 1 % magnesium chloride in 100 mm-Tris-maleate buffer (pH 9·2), dewaxed sections were incubated for 2 h at room temperature in 100 mm-Tris-maleate buffer containing naphthol AS-MX phosphate (0·2 mg/ml; Sigma) dissolved in ethylene glycol monomethyl diethyl ether (Sigma-Aldrich) as a substrate, and fast red TR (0·4 mg/ml; Sigma) as a stain for the reaction product. After washing with distilled water, the sections were counterstained with methyl green nuclear counterstain (Vector Laboratories) and mounted with Kaiser's glycerol jelly.

TRAP enzyme histochemical analysis was performed on paraffin sections using a modification of a previously described protocol^(^ ^20^ ^)^. Dewaxed sections were pre-incubated for 20 min in buffer containing 50 mm-sodium acetate and 40 mm-sodium tartrate at pH 5·0. The sections were incubated for 15 min at room temperature in the same buffer containing 2·5 mg/ml of naphthol AS-MX phosphate (Sigma) in dimethylformamide as a substrate, and 0·5 mg/ml of fast garnet GBC (Sigma) as a colour indicator for the reaction product. After washing with distilled water, the sections were counterstained with methyl green and mounted with Kaiser's glycerol jelly.

### Immunohistochemical staining

Immunohistochemical staining for type I collagen (Col-I) was performed using an affinity-purified goat anti-mouse Col-I antibody (Southern Biotechnology Associates) as described previously^(^ ^17^ ^)^. Briefly, dewaxed and rehydrated paraffin-embedded sections were incubated with methanol–H_2_O_2_ (1:10) to block endogenous peroxidase activity, and then washed in Tris-buffered saline (pH 7·6). The slides were incubated with the primary antibody overnight at room temperature. After rinsing with Tris-buffered saline for 15 min, tissues were incubated with a secondary antibody (biotinylated goat anti-rabbit IgG or biotinylated goat anti-mouse IgG; Sigma). Next, the sections were washed and incubated with the VECTASTAIN Elite ABC reagent (Vector Laboratories) for 45 min. Staining was developed using 3,3′-diaminobenzidine (2·5 mg/ml) followed by counterstaining with Mayer's haematoxylin.

### Computer-assisted image analyses

After haematoxylin and eosin, histochemical, or immunohistochemical staining of sections from six mice of each group images were photographed using a Sony digital camera, adjusting the digital camera with 5·07 × 10^6^ pixels. Images of micrographs from single sections were digitally recorded using a rectangular template, and recordings were processed and analysed using Northern Eclipse image analysis software (Empix Imaging, Inc.) as described previously^(^ ^12^ ^,^ ^17^ ^,^ ^21^ ^)^. This technology was used for the determination of the trabecular bone volume relative to the total volume (BV:TV) in collagen-stained sections.

### Western blot analyses

Proteins were extracted from left femur tissues or osteoblasts from each group and quantified using Bradford protein assay kits (Bio-Rad). Protein samples (30 μg) were fractionated by SDS–PAGE and transferred to nitrocellulose membranes. Immunoblotting was performed, as described previously^(^ ^22^ ^)^ using parathyroid hormone receptor (PTHR; Sigma), IGF-1 (Millipore), Runt-related transcription factor 2 (Runx2; Bioworld), β-catenin, Wnt-5a and TRVP-6 antibodies (Santa Cruz Biotechnology, Inc.). β-Tubulin (Santa Cruz) was used as a loading control. Bands were visualised using enhanced chemiluminescence (ECL) (Amersham) and quantified by Scion Image Beta 4.02 (Scion Corporation).

### Quantitative real-time RT-PCR

RT reactions were performed using the SuperScript First-Strand Synthesis System (Invitrogen), as described previously^(^ ^21^ ^)^. Real-time RT-PCR was performed using a LightCycler system (Roche Molecular Biochemicals). Reactions included 2 ml of LightCycler DNA Master SYBR Green I (Roche), 0·25 mm of each 5′ and 3′ primer (Table 1), and 2 ml of sample (or H_2_O) to a final volume of 20 ml. Samples were amplified for thirty-five cycles with a temperature transition rate of 20°C/s for all the three steps, including denaturation at 94°C for 10 s, annealing for 5 s, and extension at 72°C for 20 s. SYBR green fluorescence was measured to determine the amount of double-stranded DNA. To discriminate specific from non-specific complementary DNA products, a melting curve was obtained at the end of each run. Products were denatured at 94°C for 30 s; the temperature was then decreased to 55°C for 15 s and increased slowly from 55 to 94°C, using a temperature transition rate of 0·188°C/s. To determine the number of target DNA copies in the samples, purified PCR fragments of known concentration were serially diluted, and served as external standards for each experiment. Data were normalised to glyceraldehyde 3-phosphate dehydrogenase levels.

**Table 1:** RT-PCR primer sequences, annealing temperatures (*T*_m_) and amplicon lengths (bp)

Genes	Primer sequences	*T*_m_ (°C)	Product size (bp)
*Runx2*	F: 5′-CAAGAAGGCTCTGGCGTTTA-3′	55	84
	R: 5′-TGCAGCCTTAAATGACTCGG-3′		
*Alp*	F: 5′-CGGGACTGGTACTCGGATAA-3′	56	157
	R: 5′-ATTCCACGTCGGTTCTGTTC-3′		
*Ocn*	F: 5′-CTTGGTGCACACCTAGCAGA-3′	55	186
	R: 5′-CTCCCTCATGTGTTGTCCCT-3′		
*Col-I*	F: 5′-TCTCCACTCTTCTAGTTCCT-3′	55	269
	R: 5′-TTGGGTCATTTCCACATGC-3′		
*CaSR*	F: 5′-AATAGCTCCACTGCCTTCCG-3′	60	123
	R: 5′-GTGCCGTAGGTGTTTCAGGA-3′		
*Rankl*	F: 5′-CCGCCTCCCGCTCCATGTTC-3′	60	257
	R: 5′-TTCCTTCTGCACGGCCCCCT-3′		
*Opg*	F: 5′-GGAACCCCAGAGCGAAATACA-3′	57	225
	R: 5′-CCTGAAGAATGCCTCCTCACA-3′		
*GAPDH*	F: 5′-ACCCCAGCAAGGACACTGAGCAA-3′	60	120
	R: 5′-AGGGCTCCCTAGGCCCCTCCTG-3′		

*Runx2*, Runt-related transcription factor 2; F, forward; R, reverse; *Alp*, alkaline phosphatase; *Ocn*, osteocalcin; *Col-I*, type I collagen; *CaSR*, Ca-sensing receptor; *Rankl*, receptor activator for nuclear factor-κB ligand; *Opg*, osteoprotegerin; *GAPDH*, glyceraldehyde 3-phosphate dehydrogenase.

### Osteoblast isolation, culture, and differentiation

To obtain purified osteoblasts, calvaria from newborn mice (postnatal days 1 and 2) were dissected from the surrounding muscle and soft tissue, and washed with PBS containing penicillin and streptomycin as described previously^(^ ^23^ ^)^. Briefly, the isolated calvaria were sequentially digested in αMEM (Gibco BRL) containing 0·25 % trypsin and 0·1 % I collagenase for 20 min at 37°C. Cells were collected by centrifugation, resuspended in the cell-culture medium (αMEM containing 10 % FBS, 2 mm-l-glutamine, 100 units/ml of penicillin/streptomycin and 50 μg/ml of ascorbic acid), and grown in six-well Petri dishes in a CO_2_ incubator at 37°C. The cell-culture medium was changed every 3–4 d, and the cells were passaged at approximately 80–90 % confluence. Third-passage osteoblasts were used in the following experiments.

### Osteoblast treatment with calcium, parathyroid hormone 1–34 fragment or PD98059

We divided osteoblasts randomly into different groups after the cells were adherent for 24 h. For the PTH 1–34 treatment group, PTH 1–34 was added at a concentration of 10^− 8^ m once every 48 h, and was kept in the media for 6 h, and then PTH 1–34 was removed and replaced by the cell-culture media, to create an *in vitro* PTH 1–34 intermittent treatment model. For the high-Ca treatment group, calcium chloride was added to a concentration of 5 mm for 48 h. For the high Ca and PTH 1–34 combined group, PTH 1–34 and calcium chloride were added to a concentration of 10^− 8^ m and 5 mm, respectively, on a 48 h cycle. For the first 6 h, the cell-culture media contained PTH 1–34 and calcium chloride. For the following 42 h, PTH 1–34 was removed, and the cells were cultured in cell-culture media containing calcium chloride alone. PD98059, a selective and reversible inhibitor of MAPK-activating enzyme, (20 μm; Tocris) was added to the high Ca and PTH 1–34 combined media as described previously. Cells were processed for 6 d, and subsequent experiments were completed.

### Osteoblast proliferation viability and alkaline phosphatase activity detection assays

Osteoblasts were used during the logarithmic growth phase, and suspended in the cell-culture medium. Cells were seeded in ninety-six-well plates at a concentration of 1 × 10^4^ cells/ml (in 100 μl media), and maintained in a CO_2_ incubator at 37°C. After adherence for 24 h, cells were randomly divided into different groups, and treated as described above. After treatment for 2, 4, 6 or 8 d, the treatment media was removed, and cells were incubated for 1·5 h in l00 μl of the cell-culture media containing 10 μl CCK-8 (Cell Counting Kit-8; Dojindo Company). We measured the absorbance value (optical density; OD) at 490 nm in a microplate reader. The experiment was repeated at least three times, and cell viability was calculated according to the following formula:
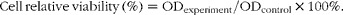



(OD_blank_ was used as the base point). ALP activity was measured using the method as described previously^(^ ^24^ ^)^.

### Mitogen-activated protein kinase assays

Third-passage osteoblast cells were treated with various reagents as described previously. After the treatment, cells were washed in PBS (20 mm-NaH_2_PO_4_, 0·9 % NaCl, pH 7·4) and lysed for 20 min with lysis buffer (20 mm-Tris–HCl, pH 7·4, 150 mm-NaCl, 0·1 % Nonidet P-40, 1 % glycerol, 0·2 mm-sodium vanadate, and a protease inhibitor cocktail tablet (Sigma)/10 ml of buffer). Samples were collected, microcentrifuged at 14 000 rpm for 5 min, and the supernatants were collected, assayed for protein, and prepared for Western blot analyses with an antibody against the active phosphorylated form of MAPK. Membranes were stripped and reprobed using polyclonal antibodies against MAPK (extracellular signal-regulated kinase (ERK)-1 and ERK-2).

### Statistical analyses

Data from image analyses are presented as means with their standard errors of the mean. Statistical comparisons were made using two-way ANOVA. For all analyses, *P*< 0·05 was considered statistically significant.

## Results

### Effects of the high-calcium diet and exogenous parathyroid hormone 1–34 on bone mineral density and bone volume in adult mice

**Fig. 1:**
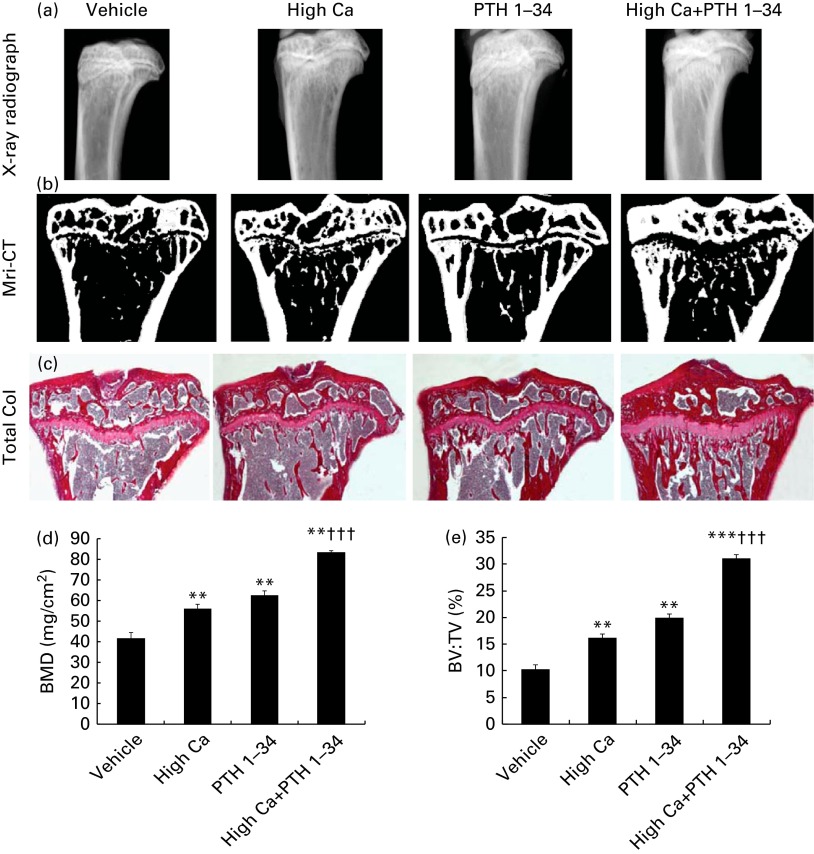
Effects of the high-calcium diet and exogenous parathyroid hormone (PTH) on bone mineral density (BMD) and bone volume in 10-week-old wild-type mice: (a) X-ray radiographs of the tibias; (b) representative longitudinal sections of the proximal ends of the tibias using micro-computed tomography (Mri-CT) and a three-dimensional reconstruction; (c) representative longitudinal sections of total collagen (Total Col) staining; (d) BMD values; (e) trabecular bone volume (BV:TV) determined in the metaphyseal regions, including both primary and secondary spongiosa. Values are means of five mice from each genotype, with their standard errors represented by vertical bars. Mean value was significantly different from that of the vehicle-treated group: ** *P*< 0·01, *** *P*< 0·001. ††† Mean value was significantly different from that of the high Ca^2+^ or exogenous PTH 1–34 treatment groups (*P*< 0·001). (A colour version of this figure can be found online at http://www.journals.cambridge.org/bjn).

To determine whether the high-Ca diet and exogenous PTH treatments influence BMD and bone volume in adult mice, the tibias were examined using X-ray radiography (Fig. 1(a)) and micro-computed tomography (Fig. 1(b)). Then, representative longitudinal sections of total collagen staining (Fig. 1(c)), BMD values (Fig. 1(d)) and trabecular bone volumes (BV:TV; Fig. 1(e)) were determined in the metaphyseal regions, including both the primary and secondary spongiosa. The results showed that BMD and trabecular bone volume were increased significantly in either the high-Ca diet group or the PTH treatment group compared with the vehicle-treated group, and were significantly higher in the combined treatment group than in the high-Ca diet or PTH treatment group (Fig. 1(a)–(e)).

### Effects of the high-calcium diet and exogenous parathyroid hormone 1–34 on osteoblastic bone formation in adult mice

**Fig. 2:**
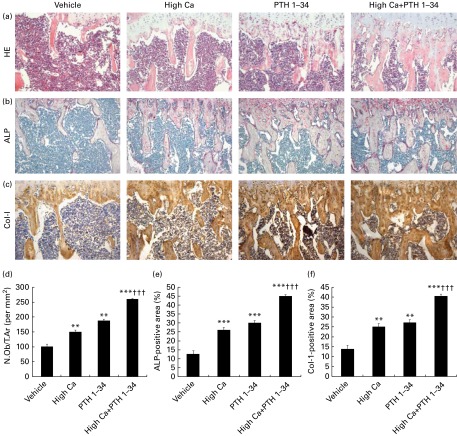
Effects of the high-calcium diet and exogenous parathyroid hormone (PTH) on osteoblastic bone formation in 10-week-old wild-type mice. Representative micrographs of the sections: (a) haematoxylin and eosin (HE) staining; (b) histochemical staining for alkaline phosphatase (ALP); (c) immunostaining for type I collagen (Col-I) from mice fed vehicle, high Ca^2+^, exogenous PTH 1–34, or high Ca^2+^ plus exogenous PTH 1–34; (d) number of osteoblasts relative to the tissue area (N.Ob/T.Ar, per mm^2^) counted in the metaphyseal regions of HE-stained tibial sections; (e) ALP-positive area as a percentage of the tissue area measured in the metaphyseal regions of tibial sections; (f) Col-I-positive area as a percentage of the tissue area measured in the metaphyseal regions of tibial sections. Values are means of five mice from each genotype, with their standard errors represented by vertical bars. Mean value was significantly different from that of the vehicle-treated group: ** *P*< 0·01, *** *P*< 0·001. ††† Mean value was significantly different from that of the high Ca^2+^ or exogenous PTH 1–34 treatment group (*P*< 0·001). (A colour version of this figure can be found online at http://www.journals.cambridge.org/bjn).

To determine whether alterations of trabecular bone volume in adult mice are associated with changes in osteoblast function, bone formation parameters were assessed by haematoxylin and eosin staining (Fig. 2(a)), histochemical staining for ALP (Fig. 2(b)), immunostaining for Col-I (Fig. 2(c)), and histomorphometric analyses for osteoblast number (Fig. 2(d)), ALP-positive area (Fig. 2(e)) and Col-I-positive area (Fig. 2(f)). The high-Ca diet treatment group displayed significantly increased osteoblast numbers, ALP-positive areas and Col-I-positive areas compared with the normal diet group. There were no significant differences between the high-Ca diet and exogenous PTH 1–34 treatment groups; however, bone formation parameters were increased significantly in the combined treatment group compared with either of these groups.

### Effects of the high-calcium diet and exogenous parathyroid hormone 1–34 on the expression of osteoblastic-related genes and proteins in adult mice

**Fig. 3:**
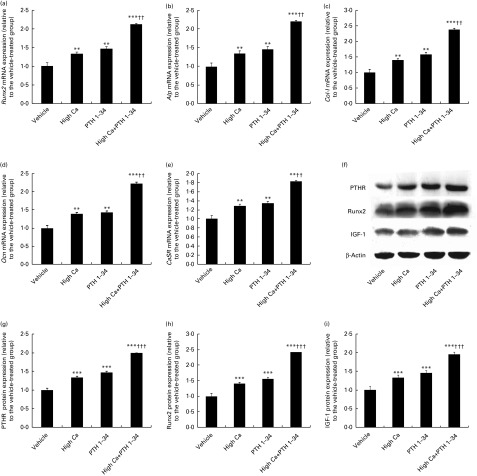
Effects of the high-calcium diet and exogenous parathyroid hormone (PTH) on the expression of osteoblastic genes and proteins in 10-week-old wild-type (WT) mice. Real-time RT-PCR and Western blot analyses were performed on the extracts from the long bones of WT mice fed vehicle, high Ca^2+^, exogenous PTH 1–34, or high Ca^2+^ plus exogenous PTH 1–34. RT-PCR of (a) Runt-related transcription factor 2 (*Runx2*), (b) alkaline phosphatase (*Alp*), (c) type I collagen (*Col-I*), (d) osteocalcin (*Ocn*) and (e) calcium-sensing receptor (*CaSR*). Protein expression of (f, g) parathyroid hormone receptor (PTHR), (f, h) Runx2 and (f, i) insulin-like growth factor 1 (IGF-1). mRNA and protein expression levels were calculated relative to glyceraldehyde 3-phosphate dehydrogenase (*GAPDH*) mRNA levels or β-actin protein levels, and expressed relative to the levels observed in WT mice. Values are means of five mice from each genotype, with their standard errors represented by vertical bars. Mean value was significantly different from that of the vehicle-treated group: ** *P*< 0·01, *** *P*< 0·001. Mean value was significantly different from that of the high Ca^2+^ or exogenous PTH 1–34 treatment group: †† *P*< 0·01, ††† *P*< 0·001.

The expression of *Runx2* (Fig. 3(a)), *Alp* (Fig. 3(b)), *Col-I* (Fig. 3(c)), osteocalcin (*Ocn*; Fig. 3(d)) and *CaSR* (Fig. 3(e)) were assessed using real-time RT-PCR in order to determine whether alterations of osteoblastic bone formation were associated with changes in the expression of osteoblastic genes and proteins. Additionally, the expression of the related proteins including PTHR (Fig. 3(f) and (g)), Runx2 (Fig. 3(f) and (h)) and IGF-1 (Fig. 3(f) and (i)) were evaluated using Western blot analyses. Consistent with the alterations observed using aforementioned histomorphometric analyses, the gene expression levels of *Runx2*, *Alp*, *Col-1*, *Ocn* and *CaSR* were significantly increased in the high-Ca diet, PTH 1–34 and combined treatment groups. Although there were no significant differences between the high Ca and exogenous PTH 1–34 treatment groups, there was an obvious increase in these expression levels in the combined treatment group compared with either of these groups. Consistent with alterations in gene expression, the protein expression levels of PTHR, Runx2, and IGF-1 were significantly increased in the high-Ca diet group compared with the normal diet group. Moreover, the levels in the combined treatment group were significantly higher than those in either the high-Ca diet or exogenous PTH 1–34 treatment group.

### Effects of the high-calcium diet and exogenous parathyroid hormone 1–34 on osteoclastic bone resorption in adult mice

**Fig. 4:**
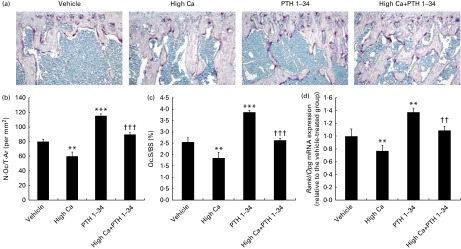
Effects of the high-calcium diet and exogenous parathyroid hormone 1–34 fragment (PTH 1–34) on osteoclastic bone resorption in 10-week-old wild-type (WT) mice. Representative micrographs of tibia sections from WT mice fed vehicle, high Ca^2+^, exogenous PTH 1–34, or high Ca^2+^ plus exogenous PTH 1–34. (a) TRAP histochemical staining; (b) number of TRAP^+^ osteoclasts relative to the tissue area (N.Oc/T.Ar, per mm^2^); (c) osteoclast surface area relative to the bone surface (Oc.S/BS, %) counted in the metaphyseal regions of tartrate-resistant acid phosphatase-stained tibial sections; (d) real-time RT-PCR performed on bone extracts for *Rankl* and *Opg* mRNA. The mRNA expression levels were calculated relative to glyceraldehyde 3-phosphate dehydrogenase (*GAPDH*) mRNA levels, and expressed relative to the expression levels of WT mice in the vehicle-treated group. The ratio of *Rankl*:*Opg* relative mRNA levels was calculated. Values are means of five mice from each genotype, with their standard errors represented by vertical bars. Mean value was significantly different from that of the vehicle-treated group: ** *P*< 0·01, *** *P*< 0·001. Mean value was significantly different from that of the high Ca^2+^ or exogenous PTH 1–34 treatment group: †† *P*< 0·01, ††† *P*< 0·001. (A colour version of this figure can be found online at http://www.journals.cambridge.org/bjn).

To determine whether alterations in osteoclast function also contribute to increased BMD and bone volume in adult mice, osteoclast number and surface were determined using histomorphometric analyses on TRAP-stained sections (Fig. 4(a)). We also examined the mRNA expression of receptor activator for nuclear factor-κB ligand (*Rankl*) and osteoprotegerin (*Opg*) in bony tissues using real-time RT-PCR, and calculated the ratio of *Rankl*:*Opg* (Fig. 4(d)). The number (Fig. 4(b)) and surface (Fig. 4(c)) of TRAP^+^ osteoclasts and the ratio of *Rankl*:*Opg* were significantly reduced in mice fed a high-Ca diet compared with those fed a normal diet. However, in the exogenous PTH treatment group, these parameters were significantly increased. No significant differences were observed in the combined treatment group compared with the normal diet group; however, compared with the high-Ca diet or exogenous PTH treatment groups, these parameters in the combined treatment group were significantly higher or lower (Fig. 4(b)–(d)).

### Effects of the high-calcium diet and exogenous parathyroid hormone 1–34 on osteoblast proliferation and differentiation in vitro

We examined the effects of Ca and PTH on osteoblast proliferation and differentiation using a cell counting kit (CCK-8; Table 2) and ALP kit (Table 3) . Osteoblast viability was increased significantly after the first cycle, and reached a peak after treatment for 6 d. However, no significant differences were observed in cell proliferation activity between the high Ca treatment and PTH treatment groups; however, viability was higher in the combined treatment group than in any of the single treatment groups (Table 2). ALP activity also increased significantly after the first cycle, and reached a peak after treatment for 6 d, and then began to decline. Although no significant differences were observed in ALP activity between the high Ca and PTH treatment groups, ALP activity was higher in the combined treatment group than in any of the single treatment groups (Table 3).

**Table 2:** Osteoblast cell viability (% of control) among the different treatment groups‡ (Mean values with their standard errors)

	Treatment time (d)
	2	4	6	8
Groups	Mean	sem	Mean	sem	Mean	sem	Mean	sem
Ca^2+^(5 mm)	126·2	4·3	151·7	5·6	174·1**	7·6	148·6	4·8
PTH 1–34 (10^− 8^ m)	131·1	3·5	162·1	5·9	186·2**	8·7	151·7	5·2
Ca^2+^+ PTH 1–34	195·8††	3·7	233·7††	6·1	272·0** ††	9·2	210·1††	5·8

PTH 1–34, parathyroid hormone 1–34 fragment.

** Mean value was significantly different from that of the vehicle-treated group at the same time point (*P*< 0·01).

†† Mean value was significantly different from that of the high Ca^2+^ or exogenous PTH 1–34 treatment group at the same time point (*P*< 0·01).

‡ Cell viability was evaluated in the high Ca^2+^, PTH 1–34 and high Ca^2+^ plus PTH 1–34 treatment groups. Osteoblast cells were treated for 2, 4, 6 or 8 d, followed by incubation for 1·5 h in 100 μl of cell-culture media containing 10 μl CCK-8 (Cell Counting Kit-8; Dojindo Company). The absorbance value (optical density; OD) at 490 nm was measured using a microplate reader.

**Table 3:** Alkaline phosphatase (ALP; U/mg protein) activity among the different treatment groups§ (Mean values with their standard errors)

	Treatment time (d)
	2	4	6	8
Groups	Mean	sem	Mean	sem	Mean	sem	Mean	sem
Control	0·0139	0·0013	0·0174	0·0014	0·0265‡	0·0019	0·0159	0·0015
Ca^2+^(5 mm)	0·0309*	0·001	0·0402*	0·0013	0·0623*‡	0·0015	0·0366*	0·0015
PTH 1–34 (10^− 8^ m)	0·0328*	0·001	0·0460*	0·0018	0·0693*‡	0·00 147	0·0430*	0·0019
Ca^2+^+PTH 1–34	0·054**†	0·0017	0·0701**††	0·00 172	0·1073**††‡‡	0·0011	0·0688**††	0·0017

Mean value was significantly different from that of the control group at the same time point: * *P*< 0·05, ** *P*< 0·01.

Mean value was significantly different from that of the high Ca^2+^ or exogenous PTH 1–34 treatment group at the same time point: † *P*< 0·05, †† *P*< 0·01.

Mean value was significantly different from that at day 2 of the same treatment group: ‡ *P*< 0·05, ‡‡ *P*< 0·01.

§ ALP activity was detected in the high Ca^2+^, PTH 1–34 and high Ca^2+^ plus PTH 1–34 treatment groups. Osteoblasts were treated for 2, 4, 6 and 8 d, and ALP activity was measured at 520 nm using a microplate reader.

### Effects of the high-calcium diet and exogenous parathyroid hormone 1–34 on the expression levels of genes and proteins during osteoblast proliferation and differentiation in vitro

**Fig. 5:**
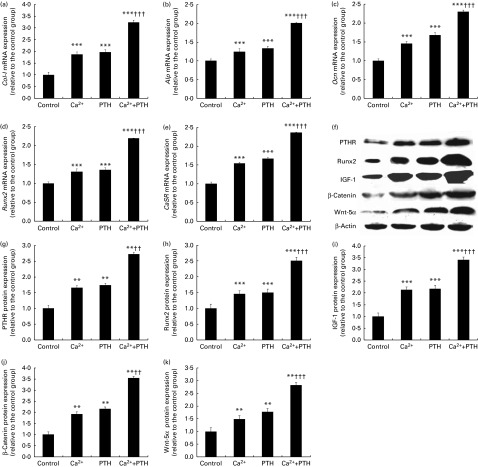
Effects of the high-calcium diet and exogenous parathyroid hormone (PTH) on the expression levels of genes and proteins during mouse calvarial osteoblast proliferation and differentiation *in vitro*. Real-time RT-PCR and Western blot analyses were performed on cell extracts from osteoblasts (third passage) treated with vehicle (1·25 mm), calcium chloride (5 mm) or PTH 1–34 (10^− 8^ m) or calcium chloride (5 mm) plus PTH 1–34 (10^− 8^ m). Gene expression levels of (a) type I collagen (*Col-I*), (b) alkaline phosphatase (*Alp)*, (c) osteocalcin (*Ocn*), (d) Runt-related transcription factor 2 (*Runx2*) and (e) calcium-sensing receptor (*CaSR*), as well as protein expression levels of (f, g) PTHR, (f, h) Runx2, (f, i) insulin-like growth factor 1 (IGF-1), (f, j) β-catenin and (f, k) Wnt-5a were determined, as described in the ‘Materials and methods’ section. The mRNA and protein expression levels were calculated relative to glyceraldehyde 3-phosphate dehydrogenase (*GAPDH*) mRNA levels or β-actin protein levels, and expressed relative to the levels of wild-type mice. Values are means of five mice from each genotype, with their standard errors represented by vertical bars. Mean value was significantly different from that of the vehicle-treated group: ** *P*< 0·01, *** *P*< 0·001. Mean value was significantly different from that of the high Ca^2+^ or exogenous PTH 1–34 treatment group: †† *P*< 0·01, ††† *P*< 0·001.

To determine the effects of Ca and PTH on gene and protein expression levels during osteoblast proliferation and differentiation, we treated third-passage mouse-derived osteoblasts as described previously. RNA was extracted, and the expression levels of *Col-I* (Fig. 5(a)), *Alp* (Fig. 5(b)), *Ocn* (Fig. 5(c)), *Runx2* (Fig. 5(d)) and *CaSR* (Fig. 5(e)) were examined using real-time RT-PCR. Protein was extracted, and the expression levels of PTHR (Fig. 5(f) and (g)), Runx2 (Fig. 5(f) and (h)), IGF-1 (Fig. 5(f) and (i)) proteins, and Wnt pathway-associated proteins (β-catenin and Wnt-5a; Fig. 5(f), (j) and (k)) were evaluated using Western blot analyses.

The expression levels of *Col-I* (Fig. 5(a)), *ALP* (Fig. 5(b)), *Ocn* (Fig. 5(c)), *Runx2* (Fig. 5(d)), and *CaSR* (Fig. 5(e)) genes and those of PTHR (Fig. 5(f) and (g)), Runx2 (Fig. 5(f) and (h)), IGF-1 (Fig. 5(f) and (i)), β-catenin and Wnt-5a (Fig. 5(f), (j) and (k)) proteins were increased significantly in the high-Ca diet, PTH and combined treatment groups. Although there were no significant differences between the high-Ca diet and PTH treatment groups, the combined treatment group had significantly higher expression levels than either the high-Ca diet or PTH treatment group.

### Effects of the high-calcium diet and exogenous parathyroid hormone 1–34 on extracellular signal-regulated kinase 1/2 phosphorylation and thrombin receptor activator peptide 6 expression in osteoblasts and the effect of PD98059 on osteoblast proliferation and differentiation

**Fig. 6:**
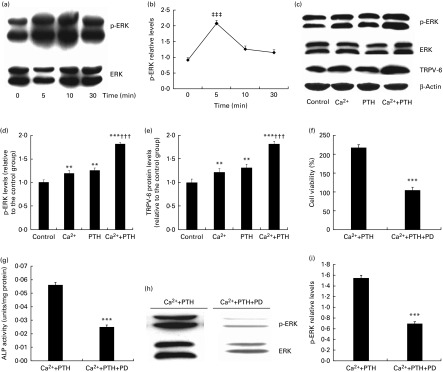
Effects of the high-calcium diet and exogenous parathyroid hormone (PTH) on extracellular signal-regulated kinase 1/2 (ERK1/2) phosphorylation and thrombin receptor activator peptide 6 (TRPV-6) expression in osteoblasts and the effect of PD98059 on mouse calvarial osteoblast proliferation and differentiation, and ERK 1/2 activation. (a) Calvarial osteoblasts from wild-type (WT) mice were treated with calcium chloride (5 mm) plus PTH 1–34 (10^− 8^ m) for 0 to 30 min and cell lysates analysed for the activation of phosphorylated ERK (p-ERK) and total ERK (ERK). (b) p-ERK levels relative to total ERK levels were assessed using densitometric analyses, and expressed relative to the levels of cells from WT mice treated for 0 min. (c) Western blot analyses of ERK-1/2, p-ERK1/2 and TRPV-6 from mouse calvarial osteoblasts (third passage) treated with vehicle (1·25 mm), calcium chloride (5 mm), PTH 1–34 (10^− 8^ m) or calcium chloride (5 mm) plus PTH 1–34 (10^− 8^ m) for 5 min. (d) p-ERK levels relative to total ERK levels were assessed using densitometric analyses and expressed relative to the levels of cells from vehicle-treated cultures. (e) TRPV-6 levels relative to total ERK levels were assessed using densitometric analyses, and expressed relative to the levels of cells from vehicle-treated cultures. (f) Cell viability, (g) alkaline phosphatase (ALP) activity, (h) Western blot and (i) p-ERK1/2 relative expression of mouse calvarial osteoblasts treated with calcium chloride (5 mm) and PTH 1–34 (10^− 8^ m) for 5 min, or calcium chloride (5 mm) combined with PTH 1–34 (10^− 8^ m) for 5 min plus pre-treatment with the ERK1/2 pathway blocker PD98059 (PD; 25 μm) for 48 h. Mean value was significantly different from that of the vehicle-treated or unblocked group: ** *P*< 0·01, *** *P*< 0·001. ††† Mean value was significantly different from that of the high Ca^2+^ or exogenous PTH 1–34 treatment group (*P*< 0·001) ‡‡‡ Mean value was significantly different from that of the other time-treatment groups (*P*< 0·001).

To determine whether the Ca and PTH treatments affected ERK1/2 phosphorylation (p-ERK1/2) and Ca channel thrombin receptor activator peptide 6 (TRPV-6) expression in osteoblasts, Western blot analyses were used to detect the relative expression of p-ERK1/2 (Fig. 6(a)–(d)). Treatment with high Ca^2+^ plus PTH 1–34 significantly induced ERK1/2 activation in osteoblasts, and p-ERK1/2 protein expression levels increased from baseline to 5 min. However, after 10 min, the levels returned to basal levels (Fig. 6(a) and (b)). Furthermore, p-ERK1/2 (Fig. 6(c) and (d)) and TRPV-6 (Fig. 6(c) and (e)) expression levels in osteoblasts were increased in the high Ca, PTH 1–34 and combined treatment groups. Although there were no significant differences in these protein expression levels between the high Ca and PTH 1–34 treatment groups, the combined treatment group was higher than any of the single treatment groups (Fig. 6(c)–(e)).

We examined the effect of the MEK inhibitor PD98059 on p-ERK1/2-mediated osteoblast proliferation and differentiation stimulated by Ca and PTH 1–34. Compared with the unblocked group, osteoblast viability (Fig. 6(f)), ALP activity (Fig. 6(g)), and p-ERK1/2 relative expression levels (Fig. 6(h) and (i)) were significantly decreased after the treatment with PD98059.

## Discussion

To validate the hypothesis that Ca and exogenous PTH 1–34 have synergistic effects on bone formation in adult mice, and to identify the related mechanisms, we fed 6-week-old male mice a normal or high-Ca diet, and subcutaneously injected them with vehicle (saline solution) or exogenous PTH 1–34 (80 μg/kg per d) for 4 weeks. We observed the role and mechanisms of high Ca and exogenous PTH 1–34 on osteoblastic bone formation using radiology, histopathology, and cellular and molecular biology techniques.

Previous research studies have found that dietary Ca supplementation can prevent osteoporosis and increase bone density in menopausal women^(^ ^25^ ^,^ ^26^ ^)^. Our previous animal studies have shown that BMD, bone volume and osteoblastic relative parameters were higher in mouse pups whose mothers were fed high-Ca diets compared with those fed a normal diet^(^ ^15^ ^,^ ^27^ ^)^. Randomised clinical trials have demonstrated that PTH can prevent the occurrence of osteoporotic fracture in patients. Many animal studies have confirmed that intermittent low doses of PTH significantly increase bone density, cortical BM and biomechanical strength^(^ ^28^ ^,^ ^29^ ^)^. In the present study, we found that mouse tibial BMD increased significantly compared with the normal diet group. Although there were no differences between the high-Ca diet and exogenous PTH treatment groups, this parameter was obviously increased in the combined treatment group. Histological analyses further confirmed that a high-Ca diet and exogenous PTH 1–34 treatment can improve bone microstructures in adult mice, and increase trabecular bone volume (BV:TV), consistent with the results of PIXImus detection. Again, there were no differences observed between the high-Ca diet and exogenous PTH treatment groups, but these parameters were increased in the combined treatment group. These results suggest that either high-Ca diet or exogenous PTH 1–34 treatment can increase adult mouse bone density, bone volume and bone formation, and synergistic effects in adult mouse bone density and bone volume were observed in the combined treatment group.

*In vivo* and *in vitro* studies have shown that increased bone formation is predominantly due to increased numbers of osteoblasts, resulting in increased osteoblast proliferation and differentiation^(^ ^30^ ^–^ ^32^ ^)^. Dvorak *et al.* ^(^ ^33^ ^)^ suggested that the effects of extracellular Ca ions on osteoblastic bone formation are mediated through CaSR, and *CaSR* knockout in osteoblasts reduced bone volume and diminished BMD. An *in vitro* osteoblast culture study^(^ ^8^ ^)^ has indicated that CaSR activation can promote osteoblast proliferation, as well as promote osteoblast differentiation and mineralisation. Moreover, high concentrations of extracellular Ca^2+^ can increase osteoblast-related gene expression and mineralised nodule formation. In the present study, we also found that *CaSR* and osteoblastic proliferation genes (*Runx2*, *Alp*, *Col-I* and *Ocn*) and proteins (Runx2 and IGF-1) were highly expressed in mice fed the high-Ca diet compared with those fed a normal diet. Studies have shown^(^ ^34^ ^,^ ^35^ ^)^ that PTH functions in osteoblasts predominantly through PTHR to activate relevant signalling pathways that inhibit osteoblast apoptosis, increase osteoblast number, promote osteoblast differentiation and then stimulate bone formation. Our recent studies have indicated that the skeletal anabolic action of PTH in neonates results from its direct action via the PTH receptor to activate protein kinase A and phospholipase C signalling pathways and to increase the osteoblast pool, as well as from its indirect action mediated via PTH-induced increases in extracellular Ca concentrations, which indirectly promotes bone formation mediated by CaSR^(^ ^15^ ^,^ ^27^ ^)^. In the present study, we found that osteoblast number and relative receptor expression levels were up-regulated and significantly higher in the combined treatment group than in any of the single treatment groups. These results suggest that high Ca (mediated by CaSR) and exogenous PTH 1–34 (mediated by PTHR) can play a synergistic role in up-regulating CaSR and PTHR expression, activating the relevant signalling pathways, promoting osteoblast proliferation and differentiation directly or indirectly, and ultimately promoting bone formation collaboratively.

In the present study, we found that the number and surface of osteoclasts and the *Rankl*:*Opg* mRNA ratio were significantly decreased in mice fed the high-Ca diet, but were increased in the PTH treatment group compared with mice fed a normal diet. This difference may be attributed to the up-regulation of blood Ca concentrations induced by a high-Ca diet that can rapidly increase the intracellular Ca^2+^ concentration of mature osteoclasts, leading to osteoclast shrinking and inhibition of bone resorption^(^ ^36^ ^)^. The up-regulation of CaSR induced by a high-Ca diet can also lead to bone resorption^(^ ^27^ ^)^. Intermittent treatment with exogenous PTH can lead to instant increases in the expression of RANKL, and stimulate osteoclast bone resorption. Moreover, PTH also down-regulated the expression of OPG, suggesting that PTH can promote osteoclast bone absorption partly by increasing the *Rankl*:*Opg* ratio^(^ ^37^ ^,^ ^38^ ^)^. Also, we found that the number and surface of osteoclasts and the *Rankl*:*Opg* mRNA ratio in the combined treatment group were unchanged compared with the normal diet group. However, these parameters were higher in the high-Ca diet treatment group and lower in the PTH treatment group, relative to the normal Ca group. These results indicate that a high-Ca diet and exogenous PTH can play a cooperative role in regulating bone resorption.

Using *in vivo* experiments, we have confirmed that a high-Ca diet and exogenous PTH 1–34 stimulate bone formation alone, and also have a cooperative role in regulating bone formation. However, the molecular mechanism of a high-Ca diet and exogenous PTH 1–34 combination influencing osteoblast proliferation and differentiation remains unclear. The Wnt signalling pathway plays an important role in bone cell function, and can directly affect the differentiation process from pluripotent progenitor cells to osteoblasts. Studies^(^ ^39^ ^,^ ^40^ ^)^ have found that PTH can increase osteoblast number by reducing the activity of an antagonist for the Wnt signal transduction pathway. Suzuki *et al.* ^(^ ^41^ ^)^ hypothesised that PTH can combine with PTHR on the surface of osteoblasts to activate protein kinase A signalling, indirectly increase Wnt signalling pathways, and stimulate osteoblast proliferation and differentiation. In addition, PTH can inhibit osteoblast apoptosis^(^ ^42^ ^,^ ^43^ ^)^. Previous studies have also found that activated IGF-1 participate in Wnt signalling pathway activation, and stimulate osteoblast proliferation and differentiation^(^ ^44^ ^)^. The present study found that β-catenin and Wnt5α expression in mouse calvarial osteoblasts were increased in the combined treatment group, as well as in either of the single treatment groups. However, no obvious changes were observed between the two single treatment groups. These results suggest that both high Ca and low-dose intermittent PTH can up-regulate β-catenin and Wnt5α expression directly or indirectly, to activate Wnt signalling pathways. These proteins are more robustly up-regulated in the combined treatment group and synergise to promote osteoblast proliferation and differentiation. However, the specific molecular mechanism of this process remains unclear, particularly regarding how high Ca and low-dose intermittent PTH 1–34 synergise to activate Wnt signalling pathways, and regulate osteoblast proliferation, differentiation, maturation and function.

ERK is the earliest confirmed kinase in the MAPK pathway that participates in the reaction of growth factors and extracellular mitogens. Yamaguchi *et al.* ^(^ ^45^ ^)^ found that Ca ions can activate the ERK/MAPK and p38/MAPK pathways through CaSR in MC3T3 E1 cells. Recently, *in vitro* studies have found that the high Ca-activated protein kinase (MAPK) pathway is dependent on CaSR for the stimulation of mouse osteoblast proliferation and differentiation^(^ ^8^ ^)^. We found that ERK1/2 phosphorylation levels reached their highest levels after 5 min of treatment in each group, and then declined. These results suggest that ERK1/2 phosphorylation may be time-dependent after the high Ca and PTH treatments. We also found that ERK1/2 phosphorylation decreased after the PD98059 treatment, and blocked osteoblast viability and ALP activity. Furthermore, high Ca and low-dose intermittent PTH also increased TRPV-6 expression, which was significantly higher in the combined treatment group than in any of the single treatment groups. The up-regulation of TRPV-6 can promote relative receptor expression, thus accelerating the influx and accumulation of extracellular Ca^2+^. Finally, the up-regulation of ERK1/2 phosphorylation promotes osteoblastic gene expression, including *Col-I*, *Alp and Ocn*, and stimulates osteoblast proliferation and differentiation.

In summary, on the basis of the present *in vivo* and *in vitro* study, we report that high Ca and PTH can stimulate osteoblast proliferation and differentiation, and stimulate osteoblastic bone formation by up-regulating the expression of CaSR, TRPV-6, PTHR and IGF-1, and activating MAPK and Wnt signalling pathways. Furthermore, high Ca combined with PTH plays a key synergised role in promoting osteoblastic bone formation by these mechanisms.
